# Working memory accuracy for multiple targets is driven by reward expectation and stimulus contrast with different time-courses

**DOI:** 10.1038/s41598-017-08608-4

**Published:** 2017-08-22

**Authors:** P. Christiaan Klink, Danique Jeurissen, Jan Theeuwes, Damiaan Denys, Pieter R. Roelfsema

**Affiliations:** 1 Vision & Cognition, Netherlands Institute for Neuroscience, Royal Netherlands Academy of Arts & Sciences, Amsterdam, The Netherlands; 2 Neuromodulation & Behaviour, Netherlands Institute for Neuroscience, Royal Netherlands Academy of Arts & Sciences, Amsterdam, The Netherlands; 3 Department of Psychiatry, Academic Medical Center, University of Amsterdam, Amsterdam, The Netherlands; 4 Department of Integrative Neurophysiology, Centre for Neurogenomics and Cognitive Research, VU University, Amsterdam, The Netherlands; 5 Department of Neuroscience, Zuckerman Mind Brain Behavior Institute, Columbia University, New York, USA; 6 Cognitive Psychology, VU University, Amsterdam, The Netherlands

## Abstract

The richness of sensory input dictates that the brain must prioritize and select information for further processing and storage in working memory. Stimulus salience and reward expectations influence this prioritization but their relative contributions and underlying mechanisms are poorly understood. Here we investigate how the quality of working memory for multiple stimuli is determined by priority during encoding and later memory phases. Selective attention could, for instance, act as the primary gating mechanism when stimuli are still visible. Alternatively, observers might still be able to shift priorities across memories during maintenance or retrieval. To distinguish between these possibilities, we investigated how and when reward cues determine working memory accuracy and found that they were only effective during memory encoding. Previously learned, but currently non-predictive, color-reward associations had a similar influence, which gradually weakened without reinforcement. Finally, we show that bottom-up salience, manipulated through varying stimulus contrast, influences memory accuracy during encoding with a fundamentally different time-course than top-down reward cues. While reward-based effects required long stimulus presentation, the influence of contrast was strongest with brief presentations. Our results demonstrate how memory resources are distributed over memory targets and implicates selective attention as a main gating mechanism between sensory and memory systems.

## Introduction

We can neither instantaneously perceive nor remember all the visual information we encounter. The mechanisms that determine which subsets of sensory information will be selected for further processing are crucial in shaping goal-directed behavior, which makes them a central topic of interest in the cognitive neurosciences. Working memory is one of those mechanisms. It is defined as the temporary (seconds) storage and manipulation of sensory information^1^. The capacity of working memory is limited, and even though the precise nature of this capacity limit is debated^2–4^, it is obvious that not all sensory information can be stored in memory and that a selection has to be made. In understanding working memory as a central aspect of cognition it is important to know what types of selection signals can influence working memory fidelity and at what stages of the memory process (encoding, maintenance, and retrieval).

Prioritization and selection of sensory information for further processing based on a combination of exogenous stimulus features and endogenous factors like task relevance and motivation can be described in terms of selective attention or “priority maps”^5–11^. The importance of both bottom-up and top-down influences on encoding of visual information is widely recognized^12–17^ making selective attention a prime candidate to selectively gate visual information into working memory^18, 19^. Indeed, previous studies have shown that attended items are more likely to be remembered than unattended or less attended items^20–26^.

The probability^23, 24, 27–33^ or precision^34, 35^ with which an object is reproduced from working memory can also be affected by cues that are presented after the stimuli have already disappeared from the screen. These ‘retro-cues’ only have an effect when they reliably indicate which memorandum will be probed^36^, suggesting a shift from a memory mode in which retention resources are distributed over multiple memorized objects to a focused mode^37^ in which irrelevant objects in memory are actively ‘forgotten’ and the relevant memory is strengthened^32, 38^. In contrast to such an all-or-nothing selection cue that eliminates the competition between individual memories for representational resources, we asked whether a redistribution of resources over multiple memory items is also possible in a graded fashion based on each item’s relative priority. We furthermore asked during which phase of working memory (encoding, maintenance, retrieval) such a priority-based selection mechanism could effectively change the precision of a memories.

Reward expectation is an important factor in goal-directed behavior and its neural signature is often similar to that of top-down selective attention^39–42^. Functionally, reward expectation is thought to increase a stimulus’ motivational salience and as a consequence attract more attention^43–48^. Studies on working memory have demonstrated influences of bottom-up stimulus salience on the probability of remembering an item from a larger array of potential targets^49, 50^ and on the precision of the memorized spatial location of objects^19, 51^. In addition, when visual items are presented sequentially, the precision with which an item is remembered depends on its position within the sequence, but also on task relevance and reward expectancy^50, 52–57^ suggesting a dynamic updating of the amount of resources assigned to each memory representation as new items are stored in memory.

In the current study, we directly compared the efficacy of reward-based priority cues in determining the fidelity of working memory for multiple targets when presented either during memory encoding, maintenance, or retrieval. Participants performed a task in which they memorized the orientation of three simultaneously presented oriented grating stimuli and reproduced the orientation of one of them on a continuous scale after a brief memory delay. Such a continuous report approach is based on recent resource models of working memory and allows inspection of the quality of working memory representations rather than just the probability that a stimulus is memorized at all^52^. To test the top-down influence of reward expectancy we used colored cues, which had been associated with different reward levels in a preceding task and indicated the amount of reward that could be earned if the associated stimulus was reproduced from memory with high enough accuracy. Crucially though, there was no indication of which stimulus memory would be probed, meaning that observers had to memorize all three targets for optimal performance. We found that reward cues only significantly influenced the accuracy of visual working memory when they were presented during the encoding phase, and not during maintenance or retrieval.

In a separate experiment, we investigated whether reward cues with predictive value need to be present during the working memory task itself to influence working memory fidelity, or whether a previously established color-reward association without predictive value would have similar effects. Indeed, we saw that a previously established color-reward association continued to influence memory accuracy for a while, even if the predictive value was no longer in place, suggesting that increased motivational salience associated with color takes some time to return to baseline in the absence of reinforcement.

Using the same basic paradigm, we compared the influence of reward-based priority signals to the effects of variations in stimulus-based salience, which was manipulated by presenting the to-be-remembered grating stimuli at different contrasts. Bottom-up stimulus salience and top-down factors like reward expectancy generally affect selective attention with rather different time-courses. The endpoints of goal-directed saccadic eye movements, for instance, have been shown to reflect a mix of stimulus salience and value based priorities that can be dissociated in time^58^. Short-latency saccades are primarily driven by stimulus salience whereas long-latency saccades are better predicted by the value information in the visual stimulus. We found a similar temporal dissociation for working memory. Stimulus salience only affected working memory precision when the stimuli were presented relatively briefly, whereas reward cues only had an effect when stimulus presentations were longer.

Together these findings demonstrate how the gate between visual representations and working memory representations is guided by a selective attention mechanism that is initially driven by stimulus salience and only later by reward predictions.

## Results

### Experiment 1: Influence of reward cues on memory accuracy

**Figure 1:**
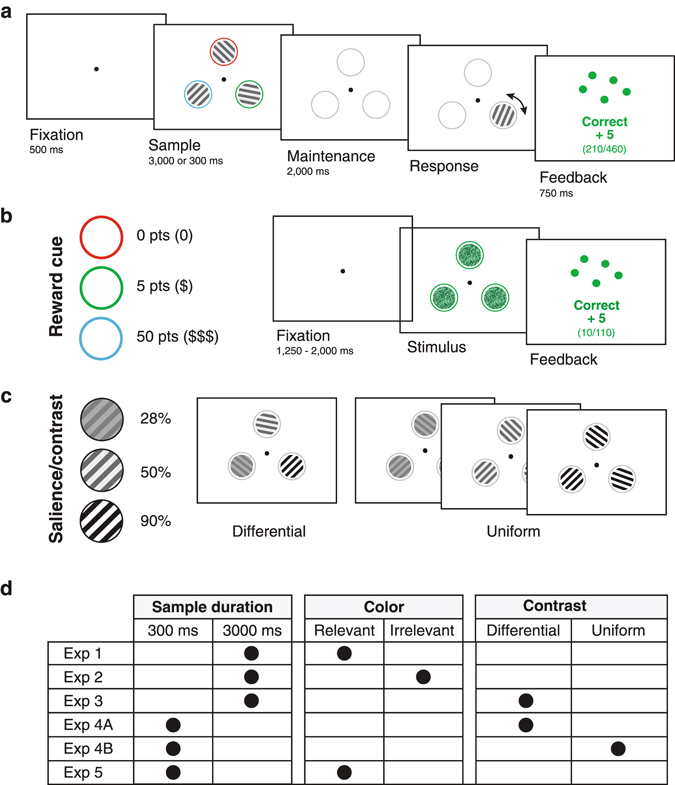
Experimental Design. (**a**) Working memory paradigm (schematic, in reality gratings were Gabors and background was gray). Trials started with 500 ms of fixation, followed by a Sample phase (3,000 ms in Experiments 1, 2, and 3; 300 ms in Experiments 4 and 5) in which three oriented Gabor patches were displayed (drawn here schematically as square wave gratings), surrounded by colored rings that were used as reward prediction cues in Experiments 1, 2, and 5. Participants remembered the orientations of the Gabors during a 2,000 ms memory maintenance phase and adjusted the orientation of a randomly selected target to match the memorized orientation in the Response phase. Feedback was provided about performance on each trial. (**b**) In Experiments 1, 2, and 5, the color of the rings around the targets was associated with a specific reward level (valid predictors in Experiments 1 and 5; no predictive value in Experiment 2). The participants first learned the association between color and reward level in a separate color-reward association task (right panel) in which they reported the color of three visual targets as quickly as possible. Feedback was provided about performance after every trial. (**c**) In Experiments 3 and 4, the contrast of the Gabors was manipulated to modulate the targets’ salience. In Experiments 3 and 4A the three targets differed in contrasts. In Experiment 4B all targets had the same contrast but the contrast level was varied across trials. (**d**) An overview of the different Experiments and their crucial parameters. Differences among experiments concern the duration of the sample phase, the presence and relevance of colored rings, and the presence and type of contrast manipulations.

**Figure 2:**
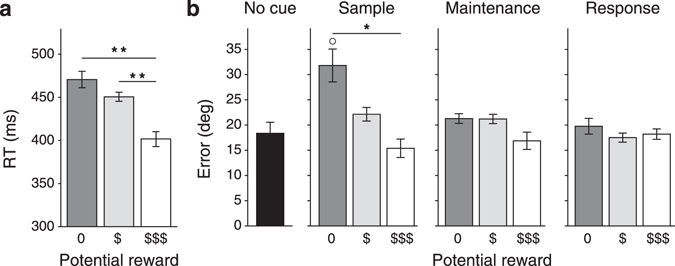
Results of Experiment 1. (**a**) Results of the color-reward association task of Experiment 1 averaged over all participants (*n* = 10). Correct color reports were generally faster for colors associated with higher rewards. (**b**) Results of the working memory task of Experiment 1 (*n* = 10). The error between the reported and the remembered orientation was smaller for stimuli surrounded by a colored ring predicting higher rewards, but only during the sample phase of the task. Error bars represent within-subjects s.e.m.’s^88^. Asterisks indicate significance levels for pairwise comparisons between reward cues (*p < 0.05; **p < 0.01). The open circle indicates a significant difference from the no cue condition (*p* < 0.05).

Experiment 1 addressed whether reward-based priority cues influence the precision of working memory in a display with multiple oriented stimuli and, if so, whether this happens during the encoding, maintenance, and/or retrieval of that memory (Fig. 1a). The experiment consisted of two phases. The first phase established the associations between colors and reward levels. The participants (n = 10) performed a reward-color association task in which they indicated, as fast as possible, which of three colors was presented (Fig. 1b). If they reported correctly within 500 ms after stimulus onset, they received a number of reward points. How many points they received (none, low, or high) depended on the color (random but stable color-reward assignments were made for each participant). Participants were generally faster in correctly reporting the high reward colors (F[2,18] = 10.98, *p* < 0.001) (Fig. 2a). Participants also made slightly fewer errors (F[2,18] = 16.23, *p* < 0.001) in this initial task for the stimuli associated with a higher reward (12.6 ± 1% [s.e.m.], 5.9 ± 0.8%, and 4.4 ± 0.7% for no, low, and high reward colors respectively). Post-hoc Tukey’s HSD tests revealed significant differences in response time between the no reward and low reward colors (*p* < 0.01), and between the no reward and high reward colors (*p* < 0.01). The same test revealed significant differences in error rate between the no reward and high reward colors (*p* < 0.01), and between the low reward and high reward colors (*p* < 0.05). These results indicate that the intended color-reward associations were indeed established.

The second phase was the working memory task. Now the participants memorized the orientation of three visual targets and reproduced the orientation of one of them. The same colors as in the reward-color association task were used in a proportion of the trials to indicate the amount of reward that could be earned if a certain target would be probed (Fig. 1a). We tested four conditions: no color cues at all, or cues during the sample, memory, or response phase of the task. The average errors in reporting the relevant orientation (inversely related to the precision of the working-memory representation) are shown in Fig. 2b. A two-way within-subjects ANOVA demonstrated a significant interaction between the independent factors ‘cueing moment’ and ‘reward level’ (F[4,36] = 7.28, *p* < 0.01) showing that the effect of reward cues (main effect: F[2,18] = 7.25, *p* < 0.02) depended on the phase of the task during which they were presented (main effect: F[2,18] = 8.73, *p* < 0.01). Additional one-way ANOVA’s for each cueing moment separately revealed that the reward prediction cues only affected the precision of working memory if they were presented during the sample phase of the working memory task (F[2,18] = 9.45, *p* < 0.01). There was somewhat of a trend towards an effect with cues presented during the maintenance phase (F[2,18] = 3.60, *p* = 0.07) and no effect with cues in the response phase of the experiment (F[2,18] = 0.92, *p* = 0.42). Post-hoc Tukey’s HSD tests revealed significant differences in accuracy between the no reward and high reward cues in the sample phase (*p* < 0.05) and between the no cue condition and the high reward sample cue (p < 0.05, all other comparisons: *p* > 0.19). This result implies that stimuli associated with a high reward during the sample phase are reproduced more reliably than stimuli associated with no reward.

Even though we only included trials in which the observers did not fixate any of the targets in the sample phase, we still performed a more extensive analysis of the eye movement data. There were no reward dependent differences in either the number of times observers fixated a target, the total time observers looked at a target, or the average distance between gaze position and the target locations in the sample and maintenance phases of the experiment (one-way ANOVA’s, all *p*’s > 0.44).

Differences in the accuracy of working memory, as measured with the average error, can be caused by a difference in the precision with which a memory is stored or by the likeliness that a memory is stored at all (guess rate). In an attempt to disentangle the relative contributions of these two factors, we fitted a probabilistic mixture model to our data that estimates the precision and guess rate from the distribution of responses^59^. Because our dataset did not have enough trials per condition for each participant to reliably fit the model on an individual basis, we pooled the data across participants and performed a bootstrapping procedure (20 times) to obtain estimates of fit reliability. This is suboptimal and any results should be interpreted with caution, but they may nevertheless provide some useful insights in the mechanisms underlying the observed effects on working memory accuracy. Indeed, the mixture model fit on the data of Experiment 1 suggests that both working memory precision and guess rates are affected by reward prediction cues in the sample phase (Supplementary Fig. S1). Compared to the no cue condition, the stimuli associated with no and low reward cues had higher guess rates, whereas the improved accuracy for high reward stimuli seems driven by a combination of improved memory precision and reduced guess rate. Cues in the maintenance phase did not affect precision, while estimated guess rates for no and low reward targets are slightly increased compared to the no cue condition.

Thus, stimuli that become associated with a high reward during the sample phase are reproduced more reliably, and with higher precision, than stimuli associated with no reward. Once the stimuli were encoded in working memory, however, reward cues had relatively little influence on memory fidelity.

### Experiment 2: Impact of previous reward associations on memory accuracy

The reward cues in Experiment 1 were established in a separate color-reward association task, but they maintained their relevance in the working memory task. Recent work, however, suggests that previously learned reward cues can influence perception and performance even when they are irrelevant for the current task^60^. Experiment 2 tested whether previous reward associations influence working memory precision. Given the weak influence of reward cues during memory maintenance and retrieval, we now only presented reward cues during the sample epoch or no cue at all. Participants first performed the color-reward association of Experiment 1. They were again faster for colors associated with higher rewards (F[2,14] = 10.68, *p* < 0.01), but now there were no significant differences in error rates between reward conditions (F[2,14] = 3.43, *p* = 0.08), which were low for all reward values (8.65 ± 1.10%, 4.49 ± 0.69%, and 5.13 ± 1.12% for no, low, and high reward colors, respectively).

**Figure 3:**
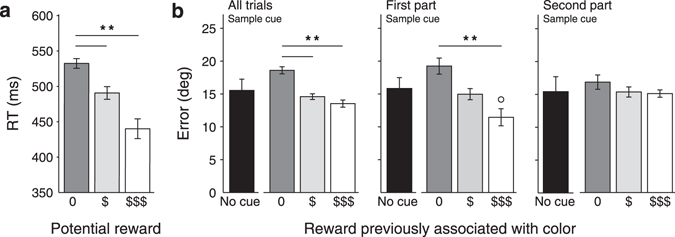
Results of Experiment 2. (**a**) Results of the color-reward association task of Experiment 2 averaged over all participants (*n* = 8). Correct color reports were generally faster for colors associated with higher rewards. (**b**) Results of the working memory task of Experiment 2 (*n* = 8). The error between the reported and the remembered orientation was smaller for stimuli surrounded by a ring in a color that previously predicted higher rewards. This effect was significant when all trials were analyzed together but strongly relied on the first half of the trials and the effect failed to reach significance in the second part of the experiment. Color cues were only presented in the sample phase, or not at all. Error bars represent within-subjects s.e.m.’s^88^. Asterisks indicate significance levels for pairwise comparisons between reward-cues (**p < 0.01). The open circle indicates a significant difference from the no cue condition (*p* < 0.05).

The colored rings were also present in the working memory task, but this time they had no predictive value for the working memory experiment. Instead, all targets were rewarded the same number of points upon correct performance (15 points). Observers were not explicitly instructed about the presence or non-predictive nature of the colored rings. A one-way within-subject ANOVA revealed a highly significant effect of cue color, with participants reproducing the orientation of the stimulus that was cued with a color that previously predicted a high reward more accurately (Fig. 3b)(F[2,14] = 18.29, *p* < 0.001; post-hoc Tukey’s HSD test, no reward color vs. low reward color: *p* < 0.01; no reward color vs. high reward color: *p* < 0.01; all other comparisons, including those with the no cue condition *p* > 0.26). Since the cueing effects were subtler in Experiment 2 than they were in Experiment 1, we wondered whether the lack of reinforcement of the color-reward association in Experiment 2 might have weakened the cueing efficacy. To investigate this scenario, we split up the trials into the first and second half of the experiment. A two-way ANOVA on working memory accuracy with factors color cue and experimental phase (first/second half) showed a main effect of color cue (F[2,14] = 8.72, *p* < 0.01), but not for experimental phase (F[1,7] = 0.12, *p* = 0.74), with a trend towards an interaction (F(2,14) = 2.74, *p* = 0.09). Because of this trend, we also performed post-hoc analysis on the influence of color cues on accuracy in the first and second phase of the experiment separately, which showed that the effect of invalid color cues was only significant in the first half of the trials (F[2,14] = 7.80, *p* < 0.01), not in the second half (F[2,14] = 0.86, *p* = 0.45). A similar split on the data from Experiment 1, where the cues predicted the amount of reward in the working memory task, did not reveal a similar dissociation (i.e. color cues during the sample phase influenced working memory in both halves of the experiment; first half: F[2,18] = 7.28, *p* < 0.01; second half: F[2,18] = 6.19, *p* < 0.02; interaction: F[2,18] = 0.21, *p* = 0.81). This finding suggests that an acquired color-reward association is only temporarily maintained as a priority cue that influences the fidelity of working memory encoding in the absence of validity. The influence of the colors faded away when reinforcement was discontinued.

We again fitted a mixture model to the pooled data to estimate the relative contributions of memory precision and guess rate to the observed working memory accuracy. In this case, the cueing effects on working memory accuracy seemed to be primarily driven by changes in memory precision, not guess rate (Supplementary Fig. S2).

### Experiment 3: Influence of stimulus contrast on memory accuracy

**Figure 4:**
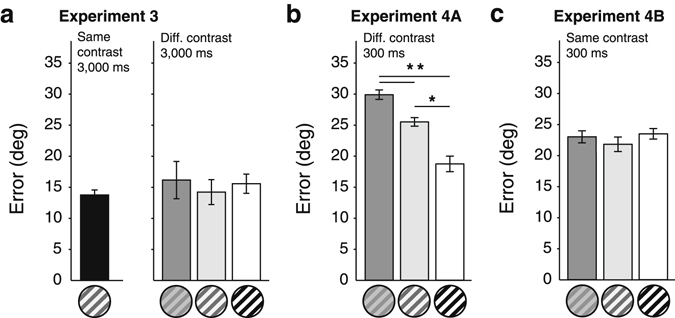
Results of Experiments 3 and 4. (**a**) Results of the working memory task of Experiment 3 averaged over all participants (*n* = 9). With a sample duration of 3,000 ms and either the same medium contrast or different contrasts for the three targets, there was no effect of salience on the precision with which target orientations were reproduced from memory. (**b**) Results of the working memory task of Experiment 4A averaged over all participants (*n* = 8). With a sample duration of 300 ms and different contrasts for the three targets, there was a significant effect of salience on the precision with which target orientations were reproduced from memory. (**c**) Results of the working memory task of Experiment 4B averaged over all participants (*n* = 8). With a sample duration of 300 ms and the same contrasts for the three targets within a trial but different contrasts between trials, there was again no effect of contrast on the precision with which target orientations were reproduced from memory. Error bars represent within-subjects s.e.m.’s^88^. Asterisks indicate significance levels for pairwise comparisons among target categories (*p < 0.05; **p < 0.01).

Reward-prediction cues provide a top-down, endogenously generated priority signal that influences memory encoding. To establish whether, in our paradigm, the precision of working memory is also influenced by low-level, exogenous factors, we manipulated the bottom-up salience^61^ of the stimuli by varying their contrast in Experiment 3 (Fig. 1c). There were two conditions. The three stimuli either all had a different contrast (low, medium, and high) during the sample phase, or they all had the same, medium contrast. Sample phases again lasted for 3,000 ms and the contrast neither predicted the amount of reward that could be earned (all targets were rewarded equally) nor the stimulus that would be probed. We observed a small yet significant effect of ‘equal contrast’ vs. ‘different contrast’ targets, but there was no effect of contrast in the ‘different contrast’ condition (Fig. 4a). The reports were on average 1.2 degrees more precise when all stimuli had the same contrast compared to when their contrasts differed. A two-way ANOVA with factors (1) equal/different contrasts and (2) contrast of the probed item, revealed a significant interaction, which suggests that displaying targets at differential contrasts affects the accuracy with which they are reproduced from memory (F[2,14] = 23.92, *p* < 0.001)(Fig. 4a). However, when we analyzed the effect of contrast for the ‘different contrasts’ condition separately, we found no significant effect of probed target contrast on memory accuracy (one-way ANOVA, F[2,14] = 0.76, *p* = 0.49). Multiple comparisons between individual conditions (including the same contrast condition) returned no significance differences either (all *p* > 0.23). This lack of a contrast-based effect on working memory was mirrored in the results of mixture model fits (Supplementary Fig. S3).

Our control analysis of eye-positions during the maintenance phase revealed that observers did not fixate targets of a particular contrast more often or longer during the sample phase, nor was their average fixation position biased towards any of the contrasts (all *p* > 0.76). Thus, at first sight, stimulus contrast seems to have only little influence on the accuracy of working memory. Combined with the results of Experiments 1 and 2, the results of Experiment 3 suggest that endogenous attention shifts induced by reward contingencies have a stronger influence on the fidelity of a memory trace than bottom-up salience. However, we wondered whether the absence of an effect of contrast might be related to the relatively long duration (3,000 ms) of the sample phase in Experiment 3.

### Experiment 4: Memory accuracy with briefly presented stimuli of different contrasts

We next examined whether the lack of an effect of contrast on the precision of working memory was caused by the relatively long sample phase of Experiment 3. The effects of stimulus salience effects are often only transient^61–63^. We therefore tested if an effect of contrast occurs when we reduce the time available for encoding the orientations into working memory by shortening the sample phase. Experiment 4 A was the same as Experiment 3 apart from the fact that the sample phase lasted for 300 rather than 3,000 ms and the contrast of the stimuli always different. Now there were clear effects of stimulus contrast on the accuracy of working memory (Fig. 4b). A one-way within-subjects ANOVA revealed that this effect was highly significant (F[2,14] = 24.02, *p* < 0.001; post-hoc Tukey’s HSD tests, low contrast vs. medium contrast: *p* < 0.01; medium contrast vs. high contrast: *p* < 0.05; low contrast vs. high contrast: *p* < 0.01). Since the average error was higher than in our previous experiments with longer sample durations we wondered whether this poorer performance at low contrast could have been caused by a difference in the quality of perception rather than by an influence of contrast on the memory encoding.

Hence, Experiment 4B investigated if an effect of contrast on perception could account for the results. We now kept the contrast of the three stimuli the same within a trial, but varied contrasts across trials, using the same contrast values as in Experiment 4A (28%, 50%, and 90%). There was no consistent effect of contrast on the precision of working memory (one-way within-subjects ANOVA; F[2,14] = 0.49, *p* = 0.62)(Fig. 4c). This result indicates that contrast acted as a priority cue when multiple stimuli had to be encoded in working memory. These differential effects of contrast on working memory accuracy were complemented by the results of mixture fits to the data of Experiment 4A and 4B (Supplementary Fig. S4). Whereas both the estimates of memory precision and guess rates depended on contrast in Experiment 4A, there was no such dependency in Experiment 4B.

To investigate the role of sample duration in more detail, we analyzed the results of Experiments 4A and 3 together in a mixed ANOVA with sample duration as a between-subjects factor and target contrast as a within-subject factor. This analysis indeed revealed a significant interaction between sample duration and contrast effects (F[2,28] = 9.51, *p* < 0.001). Thus, stimulus contrast only has a strong influence on the fidelity of a working memory if the encoding time is limited. One possible reason for this dependency on brief presentations could be that stimulus contrast influences the order of encoding, so that higher contrast stimuli are encoded before stimuli with lower contrast.

### Experiment 5: Influence of reward expectancy with briefly presented stimuli

**Figure 5:**
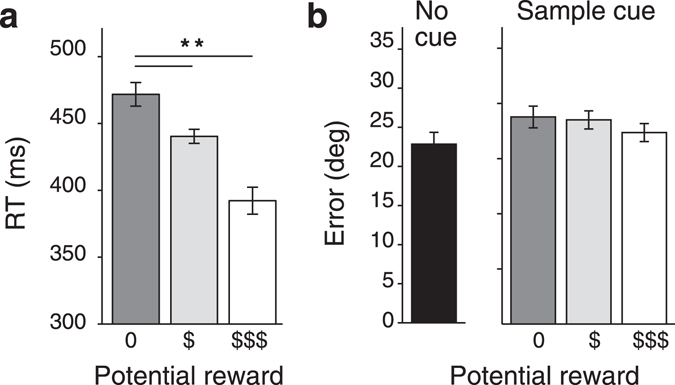
Results of Experiment 5. (**a**) Results of the color-reward association task of Experiment 5 averaged over all participants (*n* = 8). Correct color reports were generally faster for colors associated with higher rewards. (**b**) Results of the working memory task of Experiment 5 (*n* = 8). The error between the reported and the remembered orientation was not affected by colored rings in the Sample phase that predicted the potential rewards. Error bars represent within-subjects s.e.m.’s^88^. Asterisks indicate significance levels for pairwise comparisons among target categories (**p < 0.01).

We found that the influence of contrast on memory accuracy depended strongly on the duration of the encoding phase. We therefore asked if the reward-based priority influences of Experiment 1 would also depend on stimulus sample duration. Experiment 5 is essentially a repeat of Experiment 1 (including the preceding color-reward association task) but we now reduced the sample phase duration to 300 ms. Color cues were presented either in the sample phase or not all. In the color-reward association task, participants were again significantly faster for correctly responding to colors associated with a high reward (Fig. 5a) (One-way ANOVA, F[2,16] = 18.71, *p* < 0.001; post-hoc Tukey’s HSD tests, no reward vs. low reward: *p* < 0.001; low reward vs. high reward: *p* = 0.22; no reward vs. high reward: *p* < 0.001) and they made fewer errors (F[2,16] = 7.29, *p* < 0.01; average error rates were 11.11 ± 1.49%, 6.11 ± 1.24%, and 2.50 ± 1.19% for no, low, and high reward respectively). We next examined the working memory accuracy with a one-way within-subjects ANOVA. Observers were on average slightly more accurate when no color cues were presented (F[1,8] = 260.25, *p* < 0.001) but we did not observe a significant effect of reward prediction cues on memory accuracy (F[2,16] = 0.49, *p* = 0.62). Similarly, mixture model estimates of working memory precision and guess rate were hardly affected by reward cues (Supplementary Fig. S5).

We again investigated the effect of sample duration by combining the results from experiments with long and short sample durations (Experiments 1 and 5) in a mixed ANOVA with reward cue as a within-subjects factor and sample duration as a between-subjects factor. This analysis demonstrated a highly significant interaction of sample duration and reward cue effects (F[2,34] = 5.28, *p* < 0.02). Thus, the influence of the reward cues on memory accuracy are apparent at longer but not at shorter stimulus durations, whereas the effects of contrast only occur at shorter stimulus durations.

## Discussion

The current study investigated how top-down and bottom-up selection signals may prioritize the encoding, maintenance, and retrieval of multiple stimuli in visual working memory. Using color-cues associated with different levels of expected reward, we demonstrate that stimuli associated with higher expected rewards are remembered more accurately. This effect was only significant when reward-cues were presented together with the target stimuli during the encoding phase of working memory. Reward cues did however not need to have any direct predictive value for reward in the working memory task itself. Previously established color-reward associations continued to affect working fidelity for a while, but this influence disappeared over time when the color-reward association was no longer reinforced. These reward-based effects are compatible with a reward-driven increase in motivational salience. Comparing this top-down, endogenous, influence with the effect of bottom-up, exogenous, manipulations of stimulus contrast, we found that the two have fundamentally different time-courses. Exogenous cues only have an influence if sampling time is limited, suggesting that they might determine the order of encoding but not the quality of the memory traces, provided that there is sufficient time to sample all items. In contrast, endogenous cues only have an influence if sampling time is long enough as if the encoding of the most rewarding information or its strategic usage requires more time.

Previous studies have examined how stimulus salience determines the probability of remembering an item from a larger array of potential targets^49, 50^ and the precision of memorized spatial locations of objects^19, 51^. Other studies addressed the influence of task-relevance and reward-based effects on the precision of memories for the location and orientation of sequentially presented stimuli^50, 52–57^. The current comprehensive set of experiments goes beyond these previous studies by revealing the time-course of the effects of contrast and reward expectation-based on memory fidelity.

A similar time-course difference has previously been suggested to dissociate bottom-up and top-down contributions in perceptual organization^64, 65^ and the planning and execution of eye movements^58, 63^. Short-latency saccades are primarily driven by bottom-up stimulus salience, whereas long-latency saccades follow a reward-based prioritization. The fact that similar priority dynamics are found in relation to eye-movements, object recognition, and working memory, together with previous demonstrations that working memory precision is increased for future targets of a planned saccade^50, 52^, points to a central role for selective attention in working memory. Selective attention may incorporate different priority signals that become available to the system at different latencies into a general priority map for goal-directed behavior that evolves over time as priorities are dynamically updated^6, 8–11, 66, 67^. The relative stimulus priorities at the moment when visual sensory information is stored in working memory may then determine the quality with which different stimuli and their features are remembered^11^. Whereas attention may continue to interact with working memory during maintenance and retrieval^68, 69^, our results suggests that the relative precisions of multiple memories can no longer be strongly influenced when stimuli are no longer visible.

At first sight, inability of selective attention to alter the precision of working memories in the maintenance and retrieval phases may seem surprising given that several change detection studies demonstrated an influence of retro-cues on performance^23, 24, 27, 29, 37, 70–74^. A comparison between these previous studies and our work suggests that although retro-cues and attention can potentially affect the robustness of a memory (how likely it is to survive a retention interval), they may fail to alter the memory’s precision^34^.

Another recent study, however, did report effects of retro-cues on the precision of color memory^35^ and reconciling these results with our findings therefore requires an additional explanation. While reward cues during memory maintenance did not have a significant effect on memory accuracy in our study, we did observe a trend (p = 0.07) in this direction, so perhaps we simply lacked the power to reveal such an effect (Fig. 2b). On closer inspection, however, the main difference between encoding and maintenance seems to be caused by the decreased accuracy for the zero-reward target in the encoding phase relative to the no cues condition, which was not caused by retro-cues during maintenance. These results imply that memory storage resources are differentially assigned to the stimulus representations during encoding, but that they can no longer be redistributed over multiple memory stores once the stimuli have disappeared. In virtually all retro-cueing studies, including the one that reports an effect on precision^35^, cues indicated which stimulus memory would most likely be probed on any given trial. It is very well possible that the shift from a distributed memory resource mode to a focused memory mode^37^ presumably evoked by such cues does not occur in our paradigm because observers could equally likely be asked to reproduce any of the three available stimuli. This would essentially prohibit them from freeing up memory resources by ‘forgetting’ about less rewarded targets^32, 38^. Our mixture model analysis is in agreement with this interpretation as it shows that while guess rates still seem to depend somewhat on reward cues presented in the maintenance phase, memory precision is only affected by cues during the sample phase. The fact that observers do not forget a memory that, when probed, would never be rewarded with points (that directly relate to a monetary reward) furthermore suggests that giving a correct response might be intrinsically rewarding as well (performance feedback was given after each trial).

Current contrasting views on the nature of working memory primarily revolve around the dichotomy between a discrete slot capacity model and the continuous resource hypothesis^2–4, 75^. While these conceptualizations are not entirely exclusive, a thorough factorial comparison of working memory models against an extensive set of experimental data recently concluded that the predictions from the class of ‘variable precision’ models matches the existing data better^76^. The priority-driven precision modulation that we report here also supports a model in which items can be memorized with variable precision. With a limited set size of three targets, our experiments were not designed to probe the upper resource bound of working memory, but our results are compatible with a selective distribution of memory resources over visual memories. Our results with the varying contrasts demonstrate that encoding priority is a relative measure because stimulus contrast only affected memory fidelity if there were differences in stimulus contrast across simultaneously presented stimuli and not when the contrasts of all three stimuli were the same.

It is of interest to briefly consider the neural interpretation of the present prioritization effects. Neurons that encode the content of memory through the persistent firing of action potentials are a common element in many models of working memory^77, 78^. Such neurons have been shown to exist in inferior temporal and prefrontal cortex^79–81^ and the persistent activity decreases when more items have to be coded at the same time^82^. Furthermore, items that are more salient because they are associated with a higher reward or punishment cause stronger activity than items that are less salient^83^. It therefore seems likely that the stronger responses elicited by the higher contrast stimuli and of stimuli associated with a higher reward explains their competitive advantage during encoding^84^.

The comprehensive set of experiments we present here provides new insights into the differential effects of endogenous and exogenous salience on the precision of working memory for multiple simultaneously presented targets. We show that reward expectancy only significantly modulates working memory accuracy when cues are presented sufficiently long during the encoding phase of memory, when sensory codes are transformed into memory codes. Retro-cueing reward expectations after the stimuli were removed from the screen did not significantly affect precision, presumably because our task required observers to memorize all three targets and memory could therefore not switch from a distributed to a focused mode. Cues that previously predicted a reward but are currently not predictive anymore continue to affect the fidelity of working memory during encoding in a similar way, but their effectiveness gradually weakens in the absence of reinforcement, supporting the idea that reward expectancy changes the motivational salience of stimuli. The exogenous salience of stimuli influenced working memory fidelity with a fundamentally different time-course than the reward cues. They were only effective when the encoding phase was relatively short, which suggests bottom-up salience might primarily determine the order or encoding rate with which memories are stored.

A previous study specifically investigated the temporal dynamics of working memory encoding and concluded that the precision of working memory is primarily limited by the rate with which memories can be encoded into memory in parallel^49^. In these experiments, precision of memory recall increased when exposure times (sample durations) were longer. Both bottom-up and top-down cues furthermore influenced memory precision. Similar to our results with stimulus contrast, these authors show that a simple salient flash facilitated working memory precision only when sample durations were short, but that predictive cues were also effective with longer exposure durations. It is furthermore argued that predictive cues allow the reallocation of memory resources since they are also effective when they appear well after the onset of the sample array, when stimuli have presumably already been encoded into memory. Crucially though, while this reallocation effect is described as a cueing effect during memory maintenance, it must be noted that stimuli were still visible to the observer at the time of the supposed reallocation. This finding, in combination with the fact the we don’t see a similar reallocation effect when we cue during a maintenance in the absence of stimuli, suggests that such reallocation might require continuing sensory input to optimally use the reallocated memory resources.

Future working memory studies could investigate whether the salience of a stimulus indeed determines the rate with which they are encoded into memory by systematically varying the exposure duration of an array of stimuli with different contrasts. If working memory encoding is indeed a contrast-dependent process, we would expect that with increasing sample duration, an incremental number of targets are memorized at higher precision with the higher contrast stimuli benefiting from prolonged exposure before the lower contrast stimuli do. Along the same lines, if working memory encoding strategies depend on the sample durations it could be of interest to investigate whether knowledge of the upcoming sample duration is an important determinant of the effect of priority cues on working memory accuracy. In our experiments, participants knew how long the sample stimuli would be displayed since the sample were always the same within an experiment and it has recently been shown that stimulus predictability could play a role in the neural representation of working memory^85^. A future experiment in which the sample duration is unpredictably varied could further unravel the prioritization strategies that determine the precision of working memory encoding.

Finally, our data suggest that selective attention dynamically incorporates exogenous and endogenous priorities during the encoding phase of visual working memory, but that the resources that determine the precision of individual memories cannot be re-distributed over multiple targets during memory storage and retrieval.

## Material and Methods

### Stimuli

Experiments were run on a Dell PC in a dimly lit room using Matlab (the Mathworks) and the Psychtoolbox 3.0 extensions^86, 87^ under the Windows 7 operating system. Stimuli were presented on a gamma-corrected 22” Dell CRT monitor with a resolution of 1280 × 1024 pixels and refresh rate of 85 Hz. Participants were seated at a distance of 57 cm from the screen with their head positioned in a chin rest. They fixated a fixation dot throughout a trial and gave reports using a standard computer keyboard and mouse. An Eyelink 1000 eye-tracking system (SR Research) continuously monitored the position and pupil diameter of the left eye at a sampling rate of 1000 Hz in all experiments except for Experiment 2.

The details of the stimuli of the working memory task varied between experiments but the basic paradigm was always the same and consisted of four phases (Fig. 1a). A brief initial (500 ms) fixation period was followed by a sample phase in which three oriented Gabor patches were shown. Participants were instructed to remember the orientations of these three Gabor patches as precisely as they could. The sample phase could last for 3,000 ms (Experiments 1, 2, & 3) or 300 ms (Experiments 4A, 4B, & 5). It was followed by a memory maintenance phase that lasted 2,000 ms without the Gabors. In the response phase, one of the Gabor patches re-appeared with a random orientation. Participants then adjusted the orientation using the mouse to match the remembered orientation of this stimulus during the sample phase. They confirmed their answer by pressing the spacebar on the keyboard. Trials in which the reproduced orientation was within 20 degrees of the original orientation were classified as correct. After answering, feedback about performance was provided on the screen for 750 ms and a new trial commenced with a new fixation phase.

A typical run would start by calibrating the eye-tracker using a nine points calibration routine spanning the width and height of the monitor. After every 20 working memory trials participants were explicitly instructed to fixate the central fixation dot and press the space-bar. The eye position that was sampled at this moment functioned as a checkpoint to control for drift in the eye position signal over an experimental run.

Stimuli were Gabor patches with a spatial frequency of 0.5 cycles per visual degree, a Gaussian envelope with a width of 5 degrees (standard deviation of 0.83 degrees), and one of 6 possible orientations (22.5, 45, 67.5, 112.5, 135, and 157.5 degrees) that did not include the cardinal directions. Three of these stimuli were equidistantly positioned on a virtual ring around fixation with a radius of 5 degrees at the 12, 4, and 8 o’clock positions (Fig. 1a). The orientations were assigned pseudorandomly but the three targets always had different orientations. In Experiments 1, 2, and 5, Gabors had a fixed Michelson’s contrast of 50%, while in Experiments 3, 4A, and 4B the contrast was varied as part of the experimental design (see below). Stimuli were presented against a neutral gray background (50% gray with a luminance of 15.4 cd/m2) and surrounded by a thin ring (0.15 deg, diameter of 5 deg) that, depending on the experiment, could have different colors and meanings (see below).

### Participants

Participants (Exp 1: 10; Exp 2: 8, including 1 author; Exp 3: 9, including 2 authors; Exp 4A: 8, including 2 authors; Exp 4B: 8, including 2 authors; Exp 5: 8, including 2 authors) were volunteers that were paid a basic participation fee of €10/h, supplemented with a bonus depending on performance (maximally €5). This bonus was determined by the number of points they earned in the experiment as a fraction of the total number of points they could have earned with 100% correct trials. Participants ranged in age between 19 and 34 and gave informed consent before participating in these experiments. They were all healthy with normal or corrected-to-normal visual acuity. The experimental procedures were approved by the Ethics Committee from the Faculty of Social and Behavioural Sciences at the University of Amsterdam and performed in accordance with all relevant guidelines and regulations.

### Procedures

In Experiments 1, 2 and 5, the working memory task was preceded by a color-reward association task designed to prime each participant with a particular color-reward association. The same stimulus layout was used here, but instead of Gabor patches the stimuli were three static white noise patches overlaid with color masks (Fig. 1b) in one of three possible colors (red, green, or blue). The rings surrounding the stimuli had the same color as the noise patch and all three stimuli were presented with the same color. After a fixation period with a variable duration that ranged between 1,250 and 2,000 ms, the stimulus configuration was presented. Participants reported the stimulus color by pressing one of three keys on the keyboard as fast as possible. If they reported correctly, within 500 ms after stimulus onset, they received a number of reward points. The number of reward points could be 0, 5, or 50 points depending on the color that was presented. These color-reward associations were randomly assigned for each individual participant. After each trial, participants were informed about their performance on that trial with a text in the color of the preceding targets. Feedback could be ‘too slow’, ‘wrong’, or ‘correct’ and always included an update of the amount of points earned on this trial, the amount of points earned thus far, and the total amount of points that could have been earned up until that point. On correct trials, the amount of earned reward was furthermore illustrated with a number of dots drawn in the color of the preceding targets that represented the number of reward points. These dots were 0.75 degrees in diameter and randomly positioned inside an 8 × 8 degree area in the middle of the screen. The color-reward association task comprised a total of 117 trials (39 for each color).

After a brief break (~5 minutes), participants continued with a version of the working-memory task. For Experiment 1, the Sample duration was 3,000 ms. Reward-prediction cues were implemented on 75% of 432 trials using differently colored rings around the Gabor patches. One stimulus had a ring in the color that corresponded to zero reward (for that participant), one ring was in the color for a reward of 5 points, and one would indicate 50 points. These rings had no predictive value for which target would be probed. The color cues were only presented during a single phase of the task, either in the sample phase (25% of trials), the maintenance phase (25%), or in the response phase (25%). They were present for the full duration of the designated phase. During the other phases the rings were also present but all three were drawn in a neutral light gray color (65% luminance, 30.3 cd/m^2^). On the 25% of trials without reward cues the rings were neutral gray throughout the entire trial. Participants were reminded of the color-reward association every 40 trials using an instruction screen, and feedback about the potential and obtained reward was provided after every single trial.

Experiment 2 was similar to Experiment 1, but this time the colored rings were not predictive of the amount of reward participants could earn with a correct response. Instead, all target stimuli were equally rewarded with 15 points and no interspersed instruction screen were shown to remind observers of the color-reward association. In addition, cues were only presented during the sample phase or not at all. A total of 108 trials were performed by each observer.

Experiment 5 was also similar to Experiment 1, but here the sample phase only lasted for 300 ms. Reward prediction cues were presented either during the sample phase or not all, and they were again valid predictors for the amount of reward that could potentially be earned. There were 216 trials in total.

Experiments 3, 4 A, and 4B did not include a color-reward association task. The three rings around the Gabors were present for consistency, but they were always gray. The critical manipulation in these experiments was the contrast of the Gabor patches in the sample phase (Fig. 1c). In Experiments 3 and 4A, one of the targets had 28% Michelson contrast, one had 50% contrast (like in Experiments 1, 2, and 5), and one had 90% contrast. These contrasts were not predictive of which stimulus would be probed, or of how much reward could be earned (always 15 points). The test stimulus in the response phase was always shown at 50% contrast. In Experiment 4B, the contrast of all three Gabors in the sample phase was the same (28%, 50% or 90%), while the test stimulus was again shown at 50%. Sample phase duration was 3,000 ms in Experiment 3 and 300 ms in Experiments 4A and 4B. There were 108 trials in each experiment.

An overview of the different Experiments is given in Fig. 1d.

### Data Analysis

We analyzed the data of all the working memory experiments by calculating the average error in degrees between the orientation shown in the sample phase and the orientation reproduced in the response phase and comparing them within participants between stimulus conditions. For the color-reward association tasks, we discarded responses that were slower than 2,000 ms and calculated average response times and error rates (wrong color reported) for each color-reward combination. In all analyses, we used Mauchly’s sphericity’s test to test for the sphericity assumption in our ANOVA’s and applied Greenhouse-Geisser corrections when this assumption was violated. We checked for normality with Anderson-Darling tests and for equal variance with Bartlett’s test. We did not observe deviations from normality or unequal variances for the relevant comparisons, and we therefore performed multiple pairwise comparisons with Tukey’s HSD tests.

Eye-position data was analyzed in each phase of the working memory separately. While observers were instructed to fixate a dot in the middle of the screen, there was no immediate penalty for breaking fixation. Instead, we checked post-hoc when participants ignored their instructions and fixated one of the targets. We analyzed the frequency and cumulative duration of target fixations as a function of reward prediction or contrast and found no significant effects in any of the experiments. However, for the analysis of working memory accuracy we excluded any trial in which one of the targets was fixated for more than 50 ms during the sample phase, resulting in an average of 9.0 ± 3.0% of trials being discarded. We also checked for more subtle biases in covert attention by analyzing whether the average distance between fixation positions and the locations of the three targets depended on reward or contrast.

For each experiment, we fitted a probabilistic mixture model to our data in attempt to disentangle the relative contributions of memory precision and guess rates in our measure of working memory accuracy^59^ using an adaptation of code that is freely available from the Bays lab at the University of Cambridge (http://www.paulbays.com/code/JV10). This fitting procedure should ideally be done for each participant separately, but it requires more trials per condition for each participant than we obtained in our experiments. As a workaround, we fitted the model to data pooled over all observers within an experiment and bootstrapped it 20 times to get a sense of the reliability of the fits. Performing the analysis on pooled data greatly reduces its predictive power, which means that any results need to be interpreted with caution. With this caveat in mind, we did not perform any explicit statistical analysis and present the results of the mixture model analysis in a set of Supplementary Figs (S1–S5) rather than in the main manuscript.

## Electronic supplementary material

Supplementary Information
