# Supplementary material for: Working memory accuracy for multiple targets is driven by reward expectation and stimulus contrast with different time-courses
Source: Sci Rep. 2017 Aug 22;7:9082. doi: 10.1038/s41598-017-08608-4 (PMC5567292; doi:10.1038/s41598-017-08608-4)
Supplement: Supplementary file 1 — Supplementary Information [file 41598_2017_8608_MOESM1_ESM.pdf]

Supplementary Information

Working memory accuracy for multiple targets is driven by reward expectation and stimulus contrast with different time-courses

P. Christiaan Klink[1,2,3,4,*], Danique Jeurissen[1,5], Jan Theeuwes[6],
Damiaan Denys[2,3] & Pieter R. Roelfsema[1,3,4]

[1]Vision & Cognition, Netherlands Institute for Neuroscience,
Royal Netherlands Academy of Arts & Sciences, Amsterdam, The Netherlands
[2]Neuromodulation & Behaviour, Netherlands Institute for Neuroscience,
Royal Netherlands Academy of Arts & Sciences, Amsterdam, The Netherlands
[3]Department of Psychiatry, Academic Medical Center, University of Amsterdam, Amsterdam, The Netherlands
[4]Department of Integrative Neurophysiology, Centre for Neurogenomics and Cognitive Research,
VU University, Amsterdam, The Netherlands
[5]Department of Neuroscience, Columbia University, New York, USA
[6]Cognitive Psychology, VU University, Amsterdam, The Netherlandz

*Corresponding author: c.klink@nin.knaw.nl

Supplementary Figure S1

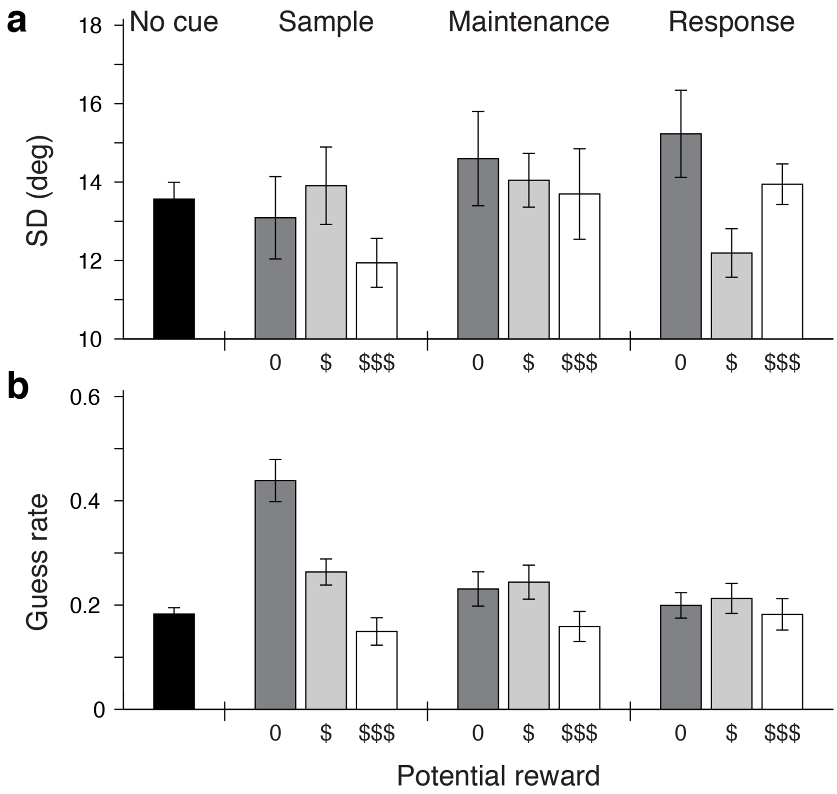

Supplementary Figure S1. Relative contributions of memory precision and guess rates in Experiment 1. A mixture model analysis[59] was performed on the data from Experiment 1 (pooled over observers) to disentangle the relative contributions of memory precision and guess rates on working memory accuracy. The mixture model fitting procedure was bootstrapped 20 times to obtain an estimate of the reliability of the result. (**a**) The standard deviation estimate (SD) of the mixture model fit reflects the precision of a memorandum (with higher SD indicating lower precision). Comparing the precision estimates for the different reward cues in the sample phase (where we saw the largest effect on accuracy, see Figure 2) with the precision in the no cue condition (black bar), it appears that the precision with which the high reward targets are reproduced ($$$, white bar) is higher than the precision with which other targets are recalled. This effect seems absent for memory cues, where each target is associated with roughly similar precision. The result for test cues (where we saw no effects on accuracy) are difficult to interpret. (**b**) The guess rate estimate of the mixture model indicates the proportion of trials (0-1) for which response angles are uniformly distributed. Comparing guess rate estimates of the cueing conditions with the estimate in the no cue condition, we can see that reward cues in the sample phase have a striking effect on the guess rate. Targets that are cued with a no or low reward color are much more likely to result in guesses. When the cues arrive in the maintenance phase, after the sample stimuli have disappeared, the increase in guess rate for the no reward condition is much reduced. For cues in the response phase the effect is similarly small. Error bars indicate standard deviation of the mean estimate from 20 bootstraps.

Supplementary Figure S2

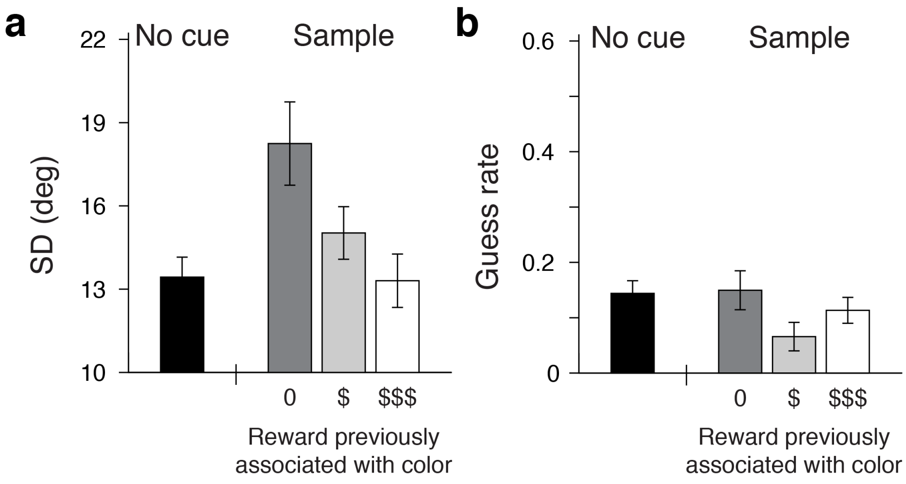

Supplementary Figure S2. Relative contributions of memory precision and guess rates in Experiment 2. A mixture model analysis[59] was performed on the data from Experiment 2 (pooled over observers) to disentangle the relative contributions of memory precision and guess rates on working memory accuracy. The mixture model fitting procedure was bootstrapped 20 times to obtain an estimate of reliability. (**a**) The precision of working memory is reduced for targets associated with a color that indicated no or low rewards in the previous task. (**b**) Guess rates are a bit lower for targets associated with a color that previously predicted a reward (either high or low). Error bars indicate standard deviation of the mean estimate from 20 bootstraps.

Supplementary Figure S3

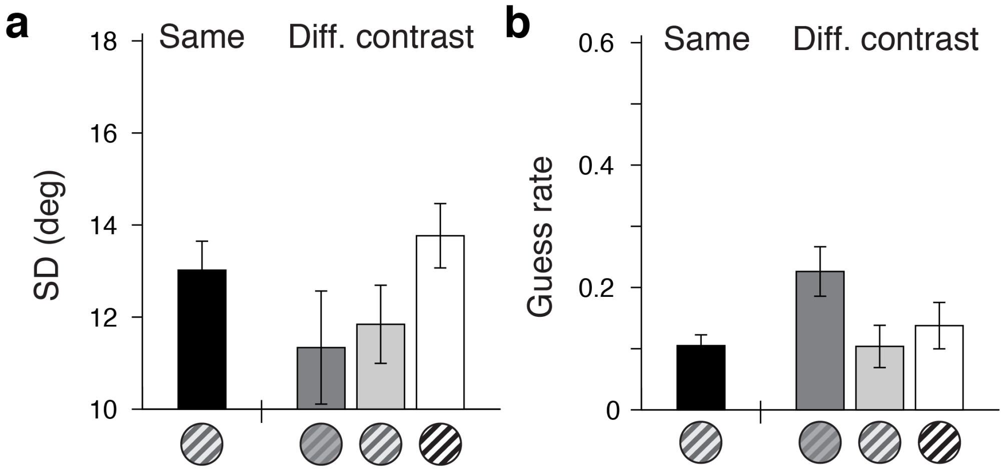

Supplementary Figure S3. Relative contributions of memory precision and guess rates in Experiment 3. A mixture model analysis[59] was performed on the data from Experiment 3 (pooled over observers) to disentangle the relative contributions of memory precision and guess rates on working memory accuracy. The mixture model fitting procedure was bootstrapped 20 times to obtain an estimate of reliability. (**a**) Working memory precision estimates do not appear to be affected much by stimulus contrast. (**b**) Guess rate estimates were a little bit higher for low contrast stimuli. Error bars indicate standard deviation of the mean estimate from 20 bootstraps.

Supplementary Figure S4

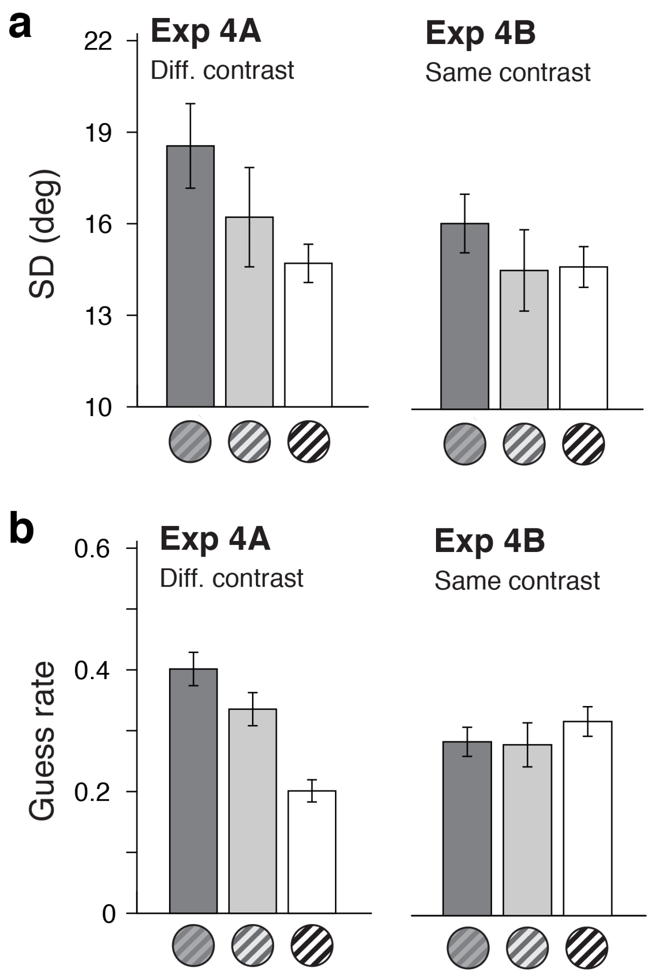

Supplementary Figure S4. Relative contributions of memory precision and guess rates in Experiment 4A and 4B. A mixture model analysis[59] was performed on the data from Experiment 4A and 4B (pooled over observers) to disentangle the relative contributions of memory precision and guess rates on working memory accuracy. The mixture model fitting procedure was bootstrapped 20 times to obtain an estimate of reliability. (**a**) In Experiment 4A, the precision of working memory varied as a function of stimulus contrast, with higher contrast stimuli being reproduced more precisely. In Experiment 4B, this effect was mostly absent. (**b**) In Experiment 4A, guess rates also varied with stimulus contrast. Guess rates were lowest for the high contrast stimuli and highest for the lowest contrast. In Experiment 4B, where the contrast of simultaneously presented stimuli was the same, but varied over trials, contrast had no effect on guess rates. In fact, guess rates are comparable with those for the middle contrast in Experiment 4A indicating that differences might indeed be driven by the relative salience of the stimuli. Error bars indicate standard deviation of the mean estimate from 20 bootstraps.

Supplementary Figure S5

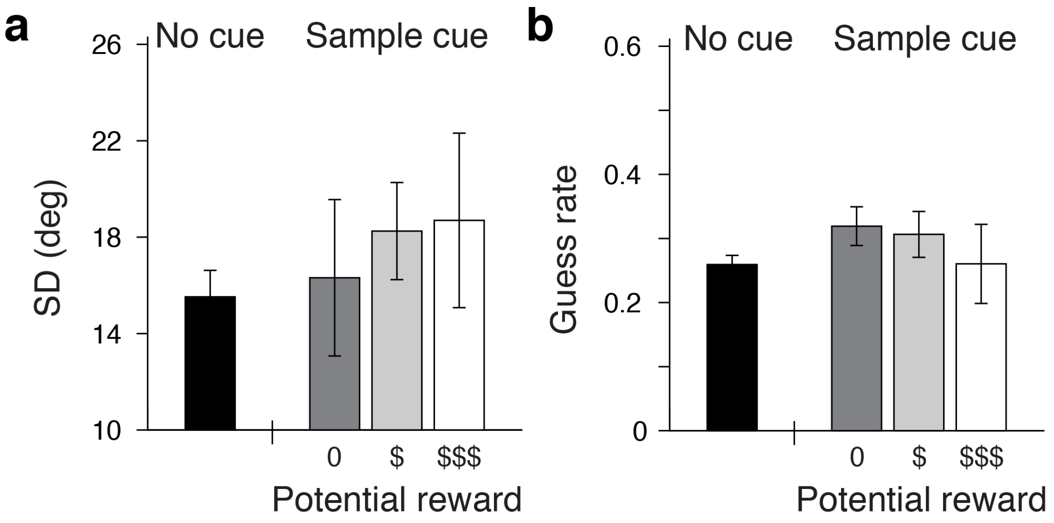

Supplementary Figure S5. Relative contributions of memory precision and guess rates in Experiment 5. A mixture model analysis[59] was performed on the data from Experiment 5 (pooled over observers) to disentangle the relative contributions of memory precision and guess rates on working memory accuracy. The mixture model fitting procedure was bootstrapped 20 times to obtain an estimate of reliability. (**a**) Precision estimates were relatively variable and appear unaffected by the reward cues. (**b**) Guess rates may be slightly higher for no and low reward predicting cues, but the effect seems very small. Error bars indicate standard deviation of the mean estimate from 20 bootstraps.
